# Treatment and Pathology of an Unusual Large Carcinoma of the Conjunctiva

**DOI:** 10.1155/2018/8461737

**Published:** 2018-04-01

**Authors:** Thomas Stahnke, Ngoy Kilangalanga, Steffi Knappe, Rudolf F. Guthoff, Andreas Erbersdobler

**Affiliations:** ^1^Department of Ophthalmology, Rostock University Medical Center, Doberaner Straße 140, 18057 Rostock, Germany; ^2^Eye Department, Hospital Saint Joseph, 322 Limete, Kinshasa, Democratic Republic of the Congo; ^3^Institute of Pathology, Rostock University Medical Center, Strempelstraße 14, 18055 Rostock, Germany

## Abstract

Carcinoma of the conjunctiva is a malignant tumor which is mostly detected and surgically treated at an early stage because of medical or cosmetic problems. Exceptions of this rule may occur in developing countries, where patients do not have access to standard medical care systems. We report the case of a conjunctival carcinoma in an African patient with an unusually late presentation. Because of local medical shortcomings, and considering the severe, transmural inflammation of anterior ocular structures, an exenteration was performed although the orbit was not deeply involved with the tumor. The strong nuclear expression of p53 indicates a major role of UV exposure in this case. A total of 16-month follow-up in this patient and larger published series suggest that the risk of metastasis is rather low under those circumstances, even with invasive tumors.

## 1. Introduction

Tumors of the outer parts of the eyeball are nearly always detected and surgically treated at an early stage. This is not only because these tumors are visible to the patient during his daily personal hygiene, but also because the ocular structures are important parts for the visual perception of an individual by other people. Tumors of the human eye often raise aesthetic concerns much earlier than medical problems. This is why clinicians, and pathologists as well, mostly have to manage incipient or early invasive tumors of the outer aspects of the eye. Exceptions of this rule may occur in developing countries, where patients do not have access to standard medical care systems.

We present a Congolese patient with advanced conjunctival carcinoma which was finally treated during a foreign medical aid program.

## 2. Case Presentation

One of the authors (R.F.G.) took part in an ophthalmologic medical aid program in Kinshasa, Democratic Republic of the Congo, which was funded by the University of Rostock in 2000. During his residence in spring 2016, a 50-year-old male Congolese requested medical attention for a large tumor of the right eyeball.

**Figure 1:**
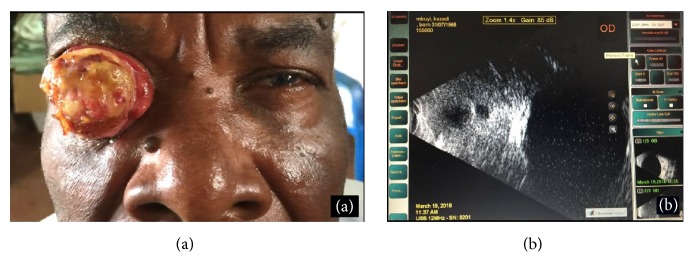
(a) Clinical aspect of the tumor. (b) Ultrasound examination.

The patient reported that his disease started in January 2015 with itching and tumefaction on his globe without pain. He consulted in one peripheral clinic and had been referred to St. Joseph hospital by an ophthalmic nurse, where he received tetracycline ointment as treatment. In February 2016 he noticed loss of vision, still without pain, fever, or loss of weight. Visual inspection of the tumor disclosed an ulcerated, soft tissue mass, which covered the whole right eyeball (Figure 1(a)).

In public, the patient hid his tumor with a pair of large sunglasses.

Physical examination showed that the tumor was fixed to the bulbar conjunctiva, totally covering the cornea. In parts, palpebral conjunctiva was also invaded. It did not seem to infiltrate deeper areas of the orbit at least by B-scan ultrasound examination (Figure 1(b)).

A malignant tumor was suspected but could not be proven because histopathologic evaluation of biopsies was not available on site. Treatment was performed by lid sparing partial exenteration of the orbit. Volume was substituted by a spherical PMMA implant with 20 mm diameter. The procedure was carried out under local anesthesia and was well tolerated by the patient.

As an alternative, volume substitution with a dermis fat graft was discussed but refused by the patient.

**Figure 2:**
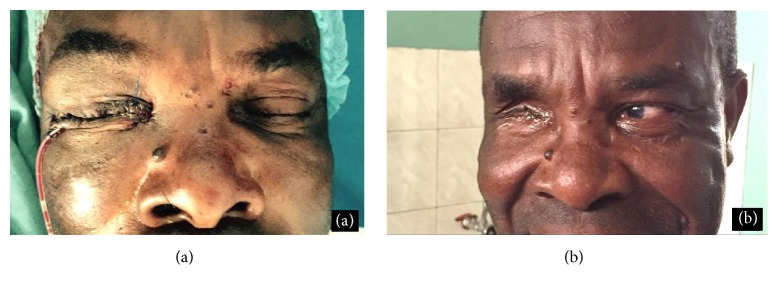
(a) Patient immediately after surgery. (b) Patient 16 months after surgery.

The patient recovered well from the surgical intervention and was pleased with the result (Figure 2(a)). Until now (16 months postoperatively) there are no signs of tumor recurrence (Figure 2(b)).

The surgical specimen was submitted to formalin and transferred to the Institute of Pathology, Rostock University Medical Center. Gross section disclosed a 2.5 cm × 2 cm × 1.5 cm mushroom-shaped mass that had fallen apart from the surface of a complete eyeball, measuring 2.5 cm in diameter with adhering parts of the eyelids and scarce orbital fatty tissue.

**Figure 3:**
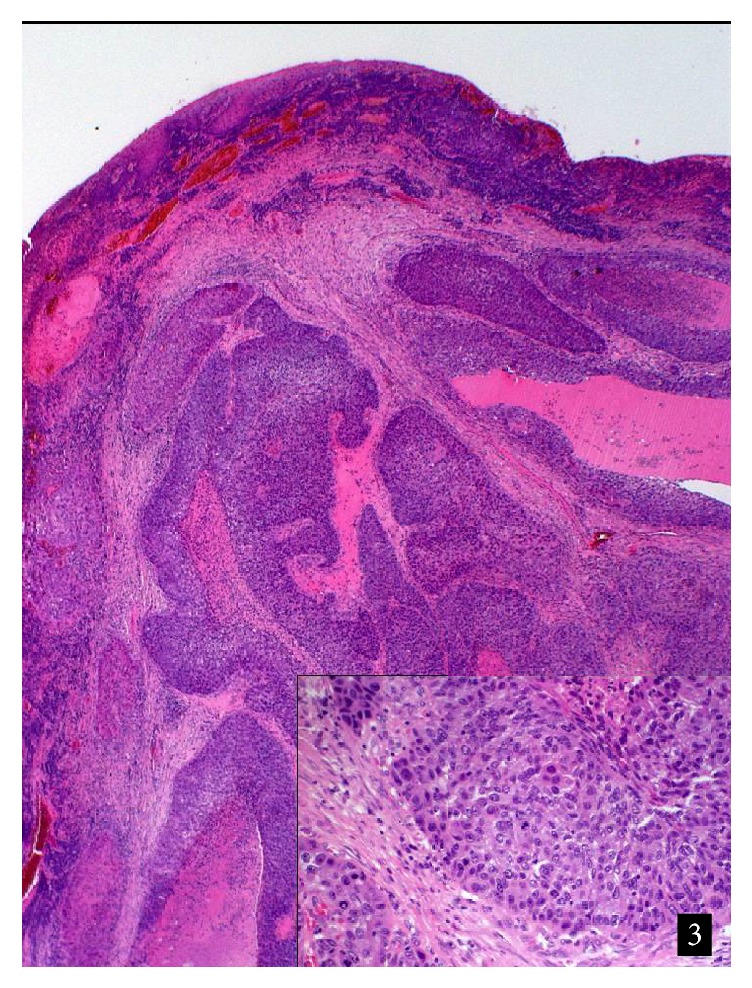
Histological aspect of the tumor. Hematoxylin & Eosin. Original magnification ×20 (inset ×100).

Histologically, the mass consisted of solid and papillary proliferation of atypical squamous epithelial cells (Figure 3).

Keratinization was not observed, but there were areas of necrosis with dense granulocytic infiltrates. At the tumor base hyperplastic nonkeratinized squamous epithelium of the conjunctiva was observed, merging into tumor proliferation. An infiltration of the subepithelial connective tissue could be demonstrated. By immunohistochemistry, the tumor cells stained with an antibody against cytokeratin (clone AE1/AE3). There was a strong, mutation-typical nuclear positivity with an antibody against p53 (clone Do7), but a complete negativity with an antibody against p16.

The fraction of proliferating cells was 20%, as detected with an antibody against Ki67 (clone Mib-1).

**Figure 4:**
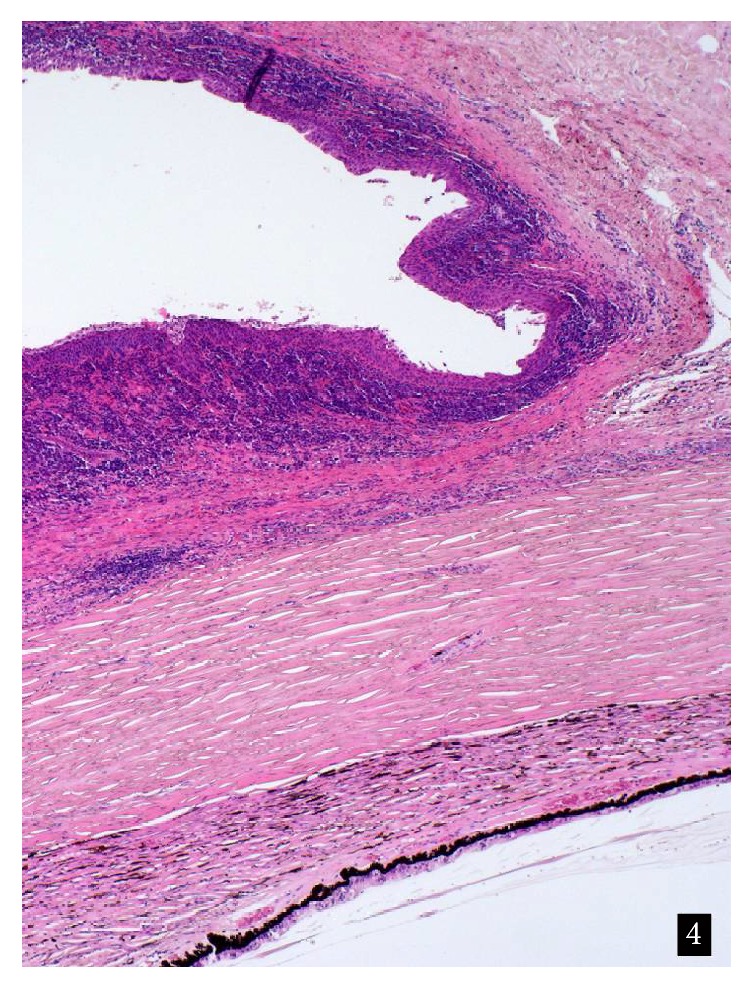
Histological aspect of the bulbar and palpebral conjunctiva. Hematoxylin & Eosin. Original magnification ×20.

Microscopic evaluation of the eyeball showed severe, chronic, lymphoplasmacellular inflammation of the bulbar conjunctiva, encroaching upon the adjacent palpebral conjunctiva (Figure 4).

There was focal acanthosis and squamous metaplasia, but no dysplasia. The cornea was involved in the inflammatory process and displayed neovascularization and an ulcerative, nearly transmural keratitis.

## 3. Discussion

Carcinoma of the ocular conjunctiva is a rare disease in Europe and North America, with mean incidence rates for males of 0.05 and 0.08 per year and 100.000 persons, respectively. It is much higher in Africa, where incidence rates range from 1.00 to 3.75 per year, per 100.000 persons [1, 2]. Solar ultraviolet (UV) exposure is believed to be the major risk factor for the development of conjunctival cancer [1, 3, 4]. Other pathogenetic factors are infections with high-risk papillomavirus (HPV) and human immunodeficiency virus (HIV) [3–5]. In the present case there was no known HIV-infection and no evidence of an overt AIDS. However, serological tests had not been performed. The negativity of tumor cells for p16 by immunohistochemistry clearly argues against an infection with high-risk HPV. On the other hand, the strong nuclear expression of p53 indicates a mutation in the TP53 gene, which is typical for an UV induced dysplasia-carcinoma sequence on the ocular surface [4]. The limbus corneae is the location which is especially vulnerable to UV induced damage and most preneoplastic lesions and small carcinomas are located at this site [4]. The exact origin of the large exophytic tumor of the patient presented in this report cannot be determined.

Histologically most epithelial neoplasia of the ocular surface are squamous cell carcinomas. Other entities include spindle cell carcinoma, mucoepidermoid carcinoma, and basal cell carcinoma [6–9]. The majority of small tumors are well differentiated squamous cell carcinomas with keratinization, but poorly differentiated cancers do occur, especially in large tumors. The present case is unique for its late medical attention, resulting in a very large, disfiguring tumor. Despite its size, the tumor only invaded the subepithelial connective tissue of the bulbar conjunctiva, but not the dense collagenous tissue of the sclera. There was also only minimal horizontal tumor spread. The eyelids were severely inflamed, but free of invasive cancer or dysplasia. An infrequent invasion of deeper ocular structures and an accompanying conjunctivitis have also been described in larger series of this tumor entity [10]. This limited invasive propensity resulted in a local tumor control in the present case, which was not necessarily anticipated before surgery. For this reason, because of local medical shortcomings and considering the severe, transmural inflammation of anterior ocular structures, an enucleation had been performed although the orbit was not involved with the tumor in retrospect. Long-term follow-up in this patient is not possible, but larger series suggest that the risk of metastasis is rather low, even with invasive tumors [10]. Furthermore, there were no signs of sympathetic ophthalmia, a complication recently described in another case report [11].

In summary, we report the case of a conjunctival cancer in an African patient with an unusually late presentation and a nevertheless successful local surgical control with an acceptable aesthetic outcome.
